# Alteration in temporal-cerebellar effective connectivity can effectively distinguish stable and progressive mild cognitive impairment

**DOI:** 10.3389/fnagi.2024.1442721

**Published:** 2024-08-29

**Authors:** Chen Xue, Darui Zheng, Yiming Ruan, Wenxuan Guo, Jun Hu, for the Alzheimer’s Disease Neuroimaging Initiative

**Affiliations:** Department of Radiology, The Affiliated Brain Hospital of Nanjing Medical University, Nanjing, China

**Keywords:** stable mild cognitive impairment, progressive mild cognitive impairment, degree centrality, directed functional connectivity, resting-state functional MRI

## Abstract

**Background:**

Stable mild cognitive impairment (sMCI) and progressive mild cognitive impairment (pMCI) represent two distinct subtypes of mild cognitive impairment (MCI). Early and effective diagnosis and accurate differentiation between sMCI and pMCI are crucial for administering targeted early intervention and preventing cognitive decline. This study investigated the intrinsic dysconnectivity patterns in sMCI and pMCI based on degree centrality (DC) and effective connectivity (EC) analyses, with the goal of uncovering shared and distinct neuroimaging mechanisms between subtypes.

**Methods:**

Resting-state functional magnetic resonance imaging combined with DC analysis was used to explore the functional connectivity density in 42 patients with sMCI, 31 patients with pMCI, and 82 healthy control (HC) participants. Granger causality analysis was used to assess changes in EC based on the significant clusters found in DC. Furthermore, correlation analysis was conducted to examine the associations between altered DC/EC values and cognitive function. Receiver operating characteristic curve analysis was performed to determine the accuracy of abnormal DC and EC values in distinguishing sMCI from pMCI.

**Results:**

Compared with the HC group, both pMCI and sMCI groups exhibited increased DC in the left inferior temporal gyrus (ITG), left posterior cerebellum lobe (CPL), and right cerebellum anterior lobe (CAL), along with decreased DC in the left medial frontal gyrus. Moreover, the sMCI group displayed reduced EC from the right CAL to bilateral CPL, left superior temporal gyrus, and bilateral caudate compared with HC. pMCI demonstrated elevated EC from the right CAL to left ITG, which was linked to episodic memory and executive function. Notably, the EC from the right CAL to the right ITG effectively distinguished sMCI from pMCI, with sensitivity, specificity, and accuracy of 0.5806, 0.9512, and 0.828, respectively.

**Conclusion:**

This study uncovered shared and distinct alterations in DC and EC between sMCI and pMCI, highlighting their involvement in cognitive function. Of particular significance are the unidirectional EC disruptions from the cerebellum to the temporal lobe, which serve as a discriminating factor between sMCI and pMCI and provide a new perspective for understanding the temporal-cerebellum. These findings offer novel insights into the neural circuit mechanisms involving the temporal-cerebellum connection in MCI.

## Introduction

Mild cognitive impairment (MCI), the preclinical stage of Alzheimer’s disease (AD), is a transitional and intermediary cognitive phase between normal aging and AD (Jessen et al., 2014a; Jessen et al., 2014b). Approximately 10–15% of MCI cases progress to AD annually, compared with only 1–2% of healthy individuals (Jessen et al., 2014b; Petersen et al., 2014). Given AD’s irreversible and progressive neurodegenerative nature, the in-depth study of MCI is of paramount importance. MCI is a heterogeneous condition; not all patients with MCI will inevitably transition to AD during their lifetime, as some may remain relatively stable, and a small subset may even regain normal cognitive function after prolonged follow-up (Mitchell and Shiri-Feshki, 2009; Petersen et al., 2014). According to the outcomes, MCI is classified into stable MCI (sMCI), which maintains MCI status, and progressive MCI (pMCI), which advances into AD (Pereira et al., 2016). The underlying neural mechanisms that govern the progression from MCI to AD remain unclear. Consequently, understanding the neuroimaging mechanisms of sMCI and pMCI is crucial for predicting MCI outcomes and enabling early and timely intervention.

In recent years, resting-state functional magnetic resonance imaging (fMRI) studies have suggested AD as a potential classic disconnection syndrome (Wang et al., 2007; Gu et al., 2020). This means that the changes in brain function in AD are not confined to the changes in a single brain area, but rather involve the changes in the whole brain network (Costumero et al., 2020). Similarly, MCI involves alterations and disruptions in the entire brain network (Xue C. et al., 2019; Xue et al., 2020). The brain connectivity patterns from the metabolic network have demonstrated decreasing inter-and intra-hemispheric connections in both sMCI and pMCI (Huang et al., 2018). Malotaux et al. reported that pMCI involved significantly higher connectivity, particularly within the default mode network (DMN), than sMCI (Malotaux et al., 2023). In pMCI, DMN connectivity increased over time, and the rate of connectivity change was correlated with the rate of cognitive decline. Cai et al. reported that sMCI and pMCI had varying degrees of alterations in the executive control network (Cai et al., 2017). However, to date, few studies have investigated the alterations in whole-brain functional connectivity (FC) patterns or directional connectivity networks associated with sMCI and pMCI.

Degree centrality (DC), a metric derived from graph theory, can assess topological properties in the whole-brain functional network. The DC represents the number of direct connections (or significant suprathreshold correlation weights) of a given voxel within the voxel-linker, measuring the importance of individual nodes. This reflects the “hub” characteristic of brain functional networks (Zuo and Xing, 2014). DC provides an unbiased approach for exploring anomalies across the complete connectivity matrix of the full-brain functional connectome. It allows for the study of functional brain abnormalities at the whole-brain level without prior hypotheses (Zuo et al., 2012). This method is confirmed to have a high level of sensitivity, specificity, and test–retest reliability. It has been utilized to examine the neurobiological mechanisms underlying brain network alterations in various neurological disorders including the AD spectrum and Parkinson’s disease (Lou et al., 2015; Zhou J. et al., 2021; Shan et al., 2023).

Furthermore, FC solely depicts interactions between distinct brain regions, whereas effective connectivity (EC) delves into the direction and intensity of information flow among these regions, providing a deeper understanding of the interaction patterns of different brain regions (Friston et al., 2013; Huang et al., 2021). EC-based findings align more closely with actual brain function mechanisms (Friston et al., 2013). Granger causality analysis (GCA), an approach for assessing EC, models interactions between significantly distinct brain regions (Liao et al., 2010). In recent years, this time-series analysis technique has played a significant role in fMRI causal modeling studies of brain regions using fMRI (Liao et al., 2010; Wang et al., 2017). GCA does not presuppose theoretical assumptions about the existence and direction of influence between any two regions. Xue et al. explored the changes of EC between the hippocampus and other brain regions in MCI and AD, and found abnormalities in the transmission and reception of information in the hippocampus (Xue J. et al., 2019). Yu et al. used GCA to explore the changes of EC of triple networks in aMCI and AD, and found that the EC (excitatory and inhibitory) obtained from GCA could distinguish AD and amnestic MCI (Yu et al., 2019). However, most of the previous studies were based on predetermined assumptions and focused on EC of single brain regions and networks, while EC analysis based on whole brain FC was rare in AD spectrum analysis. Therefore, by combining DC and GCA, an evaluation of abnormally FC in the brain network of sMCI and pMCI can yield a comprehensive and detailed understanding of their brain network changes, providing a more holistic insight into the pathophysiological processes of AD.

In this study, we aimed to delineate the intrinsic dysconnectivity pattern within whole-brain functional networks among patients with sMCI and pMCI. Initially, we employed DC to identify the brain regions manifesting altered FC within the complete brain networks of sMCI and pMCI. Subsequently, we utilized seed-based GCA to analyze EC, thereby comprehending the causal relationships of these alterations. Furthermore, we explored the links between the altered DC and EC indices and cognitive function in individuals with sMCI and pMCI. Our hypothesis posits that the EC attributes of brain nodes, as determined by DC, can evaluate the central neural mechanisms associated with the characteristics of sMCI and pMCI, potentially aiding clinical diagnosis and early intervention.

## Materials and methods

### Participants

The research data used for our study were sourced from the Alzheimer’s Disease Neuroimaging Initiative (ADNI) database.^1^ ADNI is a longitudinal multiterm study designed to develop biomarkers for early detection and monitoring of AD, encompassing clinical, imaging, genetic, and biochemical aspects. In the ADNI dataset, there are more than 400 MCI subjects scanned at screening time. After the baseline scan, follow-up scans were acquired every 3, 6 or 12 months for up to 84 months. The present investigation selected a 4-year period as the timeframe for monitoring the transition of MCI. pMCI participants were defined as participants who transitioned from MCI to AD within 4 years, while sMCI participants were defined as participants who maintained their MCI status for at least 4 years (Thung et al., 2016; Chen et al., 2023). Healthy control (HC) participants were included if they maintained HC status for a minimum of 4 years. More detailed inclusion and exclusion criteria for HC and MCI individuals can be found on the ADNI website.^2^

The current rs-fMRI dataset (*n* = 170) consisted of 96 HC participants, 42 patients with sMCI, and 32 patients with pMCI. Among them, 16 were excluded because of excessive head motion (cumulative translation or rotation >3.0 mm or 3.0^0^). Finally, the study analyzed 154 participants, including 82 HC, 41 sMCI, and 31 pMCI individuals.

### Ethics approval and consent to participate

Ethical approval for the ADNI study was granted by the institutional review committees of all participating institutions. Participants or their authorized representatives provided written informed consent (adni.loni.usc.edu).

### Neuropsychological assessment

The episodic memory (EM) and executive function (EF) were calculated according to the model provided by the ADNI website (see text footnote 1). The details regarding EM and EF were provided in Supplementary material.

### MRI data acquisition

Detailed scanning information can be obtained from http://adni.loni.usc.edu/wp-content/uploads/2010/05/ADNI2/MRI/Training-Manual-FINAL.pdf and http://adni.loni.usc.edu/wp-content/uploads/2017/07/ADNI3-MRI-protocols.pdf.

### Functional data preprocessing

Preprocessing of fMRI data was performed using Data Processing and Analysis for Brain Imaging^3^ software in MATLAB 2013b.^4^ The details regarding image preprocessing were provided in Supplementary material.

### DC analysis

Voxel-wise DC calculations were performed using DPABI software on preprocessed data to assess network centrality, capturing FC features of neural network nodes. The details regarding DC calculation were provided in Supplementary material.

A one-way analysis of variance (ANOVA) within the gray matter mask was performed using DPABI to compare the differences in DC across the sMCI, pMCI, and HC groups, with age, sex, years of education, and gray matter volume as covariates. Nonparametric permutation testing was performed with 1,000 iterations. The significance threshold was set at a level of *p* < 0.05, using threshold-free cluster enhancement (TFCE) combined with family-wise error (FWE) correction, and a cluster size of >100 voxels (2,700 mm^3^). Post-hoc comparisons were conducted using a two-sample *t*-test with the mask resulting from ANOVA, with age, sex, years of education, and gray matter volume as covariates. The threshold was set at a *p* < 0.05 with TFCE-FWE correction and a cluster size of >20 voxels (540 mm^3^).

### GCA

To explore directionality effects, GCA was employed to assess changes in EC. Based on the results of the DC analysis, we selected regions of interest (ROIs), that is, the brain regions with significant alterations in the ANOVA. All ROI coordinates were in the MNI space. EC was analyzed using a resting-state hemodynamic response function^5^ and was used to analyze EC.

In this study, the seed time series of the ROIs was defined as the seed time series x of the ROI, while the time series y represented the whole-brain voxel time series. The linear direct effects of x on y (Fx → y) and y on x (Fy → x) were calculated for each voxel in the brain. Consequently, two Granger causality maps were generated for each ROI based on the impact metric for each participant. The residual-based F was normalized (F′) and converted into Z scores for each voxel (Zy → x and Zx → y). These Z scores were derived by subtracting the global mean F′ values and dividing them by the standard deviation.

For analyzing causal connectivity at a group level, mean values of the Zy → x and Zx → y maps were computed for each group. Thus, a total of six Granger causality maps was obtained, encompassing two maps for each direction (Zy → x and Zx → y) and two for each group (the ROIs included both Zy → x and Zx → y for patients with sMCI or pMCI and HC participants). These Granger causality maps were subsequently entered into DPABI software for group comparisons.

One-way ANOVA within the gray matter mask was conducted using DPABI to evaluate differences in EC across three groups (patients with sMCI and pMCI and HC participants), while considering age, sex, years of education, and gray matter volume as covariates. The significance threshold was set with *p* < 0.05 (TFCE-FWE corrected), and a cluster size of >100 voxels (2,700 mm^3^). The two-sample *t* test was used for post-hoc comparisons with the mask resulting from ANOVA, with age, sex, years of education, and gray matter volume as covariates. The significance level was set with *p* < 0.05 (TFCE-FWE corrected) and a cluster size of >20 voxels (540 mm^3^).

### Statistical analysis

Statistical Package for the Social Sciences (SPSS) software, version 22.0 (IBM, Armonk, New York, NY, USA) was employed for statistical analysis. ANOVA and the chi-square test were utilized to compare demographic and neurocognitive scales among the three groups: sMCI, pMCI, and HC. Bonferroni correction was applied for post-hoc comparisons, and a *p* value of <0.05 was considered statistically significant.

Correlation analyses were performed in SPSS, investigating relationships between altered DC and EC and cognitive domains, adjusting for age, sex, and years of education as covariates (Bonferroni corrected, *p* < 0.05).

Receiver operating characteristic (ROC) curve analysis was carried out using SPSS 25.0 to assess the sensitivity and specificity of the altered DC and EC indexes in differentiating sMCI from pMCI.

## Results

### Demographic and neurocognitive characteristics

Table 1 presents the data of demographic and neurocognitive characteristics of all participants, including 31 patients with pMCI, 41 patients with sMCI, and 82 HC participants. The HC group exhibited a significant difference in years of education compared with the pMCI and sMCI groups. As anticipated, significant differences existed in cognitive performance, with both the pMCI and sMCI groups demonstrating significantly lower EM and EF scores than the HC group (Bonferroni corrected, *p* < 0.05).

**Table 1:** Demographics and clinical measures of three groups, including pMCI, sMCI, and HC.

	HC (82)	pMCI (31)	sMCI (41)	*F* values (χ^2^)	*p*- values
Age (years)	72.68 (6.02)	72.99 (7.06)	71.46 (7.68)	0.593	0.554
Gender (F/M)	46/36	15/16	20/21	0.863	0.650
Years of education	17.01	15.61*	15.71*	5.594	0.005^ab^
MMSE	29.06 (1.39)	26.93 (1.78)***/*	27.90 (1.53)***	23.874	<0.001^abc^
MoCA	26.24 (2.71)	21.50 (3.79)***/*	23.76 (3.30)***	27.347	<0.001^abc^
RAVLT-immediate	47.64 (10.53)	29.83 (7.62)***/*	35.90 (8.73)***	44.709	<0.001^abc^
RAVLT-learning	5.95 (2.69)	3.34 (2.18)***/*	4.90 (2.68)	11.044	<0.001^ac^
RAVLT-forgetting	3.00 (2.72)	5.55 (2.31)***	4.79 (1.92)***	14.550	<0.001^ab^
ADAS11	8.20 (3.32)	13.06 (4.95)***	8.71 (4.03)***	17.740	<0.001^ac^
ADAS13	11.83 (5.31)	20.42 (7.20)***	13.84 (6.26)***	22.552	<0.001^ac^
ADASQ4	2.70 (2.15)	6.23 (2.50)***/**	4.51 (2.40)***	28.048	<0.001^abc^
LDELTOTAL	14.02 (3.87)	4.57 (3.37)***/**	7.55 (2.81)***	92.899	<0.001^abc^
TRABSCOR	69.83 (35.72)	142.41 (77.92)***/***	92.05 (36.49)	25.352	<0.001^ac^
FAQ	0.30 (1.76)	7.07 (4.71)***/***	1.87 (2.74)	61.161	<0.001^ac^
EM	1.07	−0.11***/***	0.43***	54.720	<0.001^abc^
EF	1.16	−0.08***/**	0.57**	27.259	<0.001^abc^

Numbers are given as means (standard deviation, SD) unless stated otherwise. MMSE, Mini-mental State Examination; MoCA, Montreal Cognitive Assessment; RAVLT, Rey Auditory Verbal Learning Test; ADAS, Alzheimer’s Disease Assessment Scale-Cognitive subscale; LDELTOTAL, Logical Memory Test Delayed Recall; TRABSCOR, Trail Making Test B; FAQ, Functional Activeities Questionnaire; EM, episodic memory; EF, executive function; ^a^post-hoc analyses showed a significantly group difference between pMCI and HC; ^b^post-hoc analyses showed a significantly group difference between sMCI and HC; ^c^post-hoc analyses showed a significantly group difference between pMCI and sMCI; *p < 0.05; **p < 0.01; ***p < 0.001; pMCI, progressive mild cognitive impairment; sMCI, stable mild cognitive impairment; HC, healthy control.

### Degree centrality analysis

**Figure 1:**
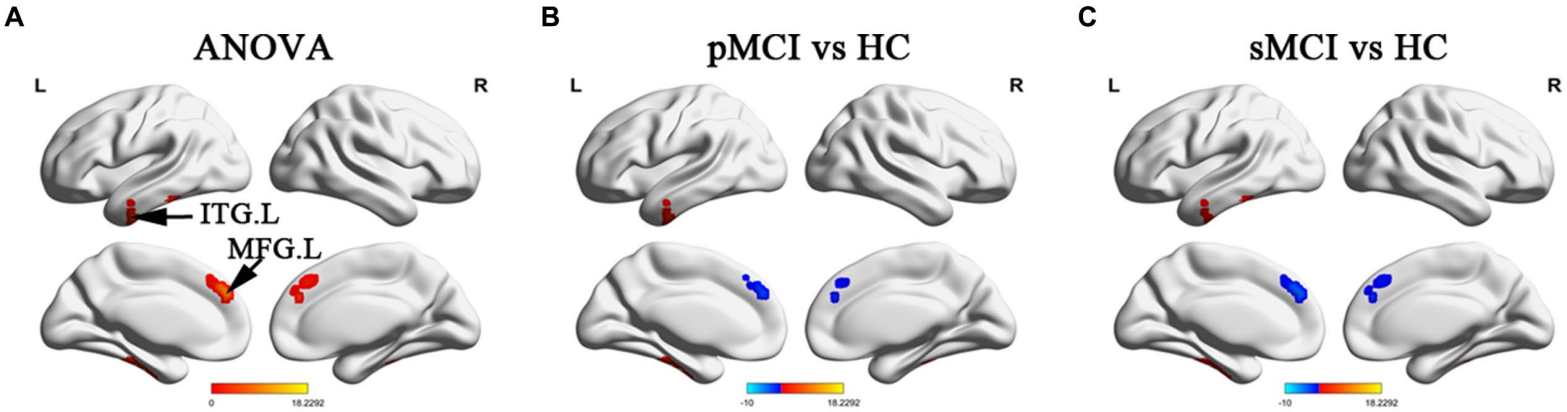
Brain regions exhibiting significant differences in degree centrality within cerebrum. **(A)** Brain regions within brain hemispheres across three groups, including sMCI, pMCI, and HC (TFCE-FWE corrected, cluster size >100, *p* < 0.05). **(B,C)** Results of post-hoc analysis in voxel-wise analysis (TFCE-FWE corrected, cluster size >20, *p* < 0.05). sMCI, stable mild cognitive impairment; pMCI, progressive mild cognitive impairment; HC, healthy control; ITG. L, left inferior temporal gyrus; MFG. L, left medial frontal gyrus.

**Figure 2:**
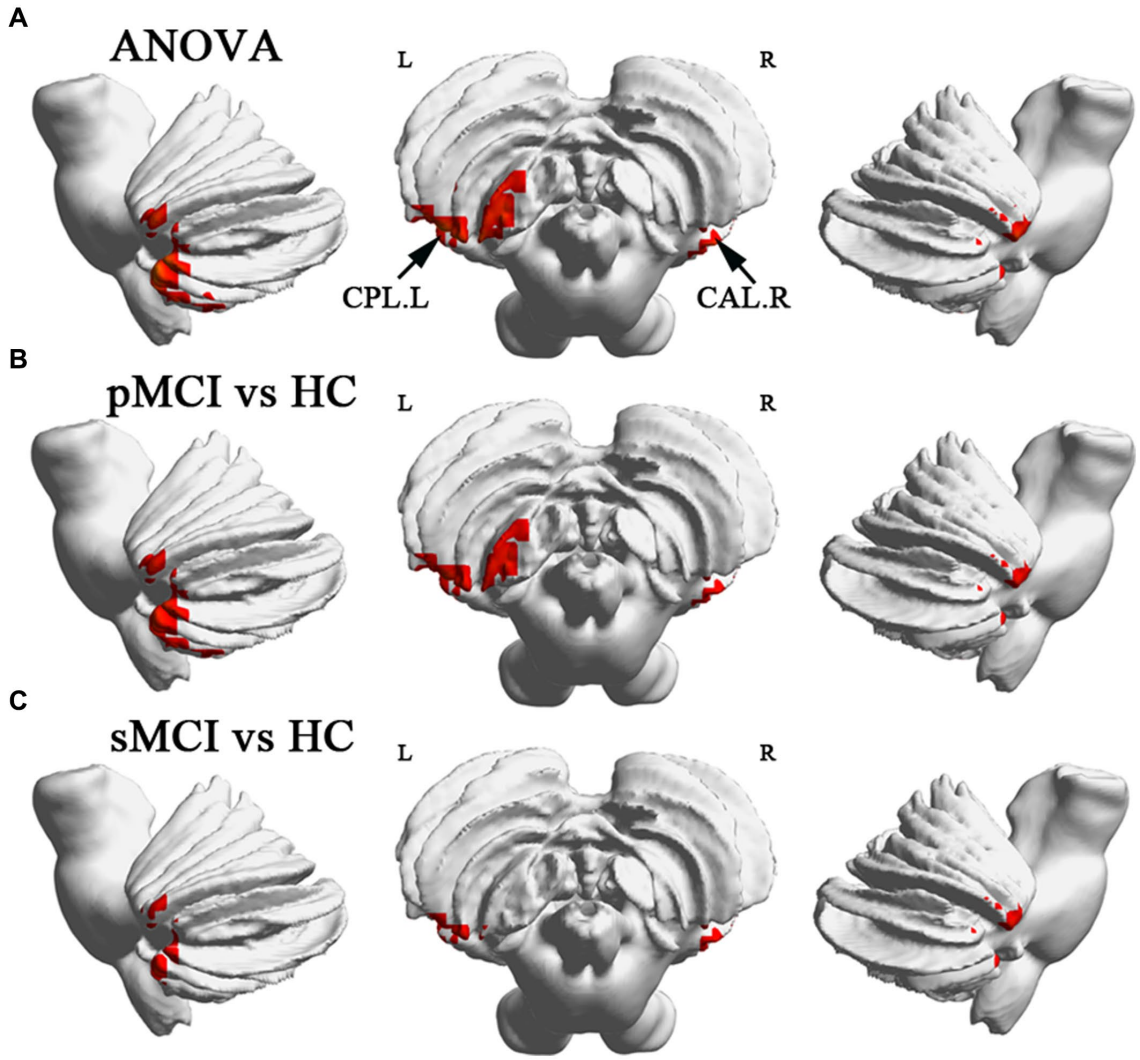
Brain regions exhibiting significant differences in degree centrality within cerebellum. **(A)** Brain regions within cerebellum across three groups, including sMCI, pMCI, and HC (TFCE-FWE corrected, cluster size >100, *p* < 0.05). **(B,C)** Results of post-hoc analysis in voxel-wise analysis (TFCE-FWE corrected, cluster size >20, *p* < 0.05). sMCI, stable mild cognitive impairment; pMCI, progressive mild cognitive impairment; HC, healthy control; CPL. L, left cerebellum posterior lobule; CAL. R, right cerebellum anterior lobule.

ANOVA showed significant alterations in DC among the groups, including the left inferior temporal gyrus (ITG), left cerebellum posterior lobe (CPL), right cerebellum anterior lobe (CAL), and left medial frontal gyrus (MFG). When compared with the HC group, the pMCI group showed increased DC in the left inferior frontal gyrus (IFG), left CPL, and right CAL and decreased DC in the MFG. Meanwhile, sMCI exhibited increased DC in the left ITG, left CPL, and right CPL and decreased DC in the MFG (TFCE-FWE corrected, cluster size of >20, *p* < 0.05). These results were obtained while accounting for age, sex, years of education, and gray matter volume (Table 2 and Figures 1, 2).

**Table 2:** The difference of degree centrality across three groups.

Region (aal)	Peak MNI coordinate			*F*/*t*	Cluster number
	*x*	*y*	*z*		
ANOVA					
L inferior temporal gyrus/cerebellum posterior lobe	−45	−18	−21	18.2292	628
R cerebellum anterior lobe	42	−36	−30	17.4254	109
L medial frontal gyrus	0	33	42	11.5542	115
pMCI vs. HC					
L inferior temporal gyrus/cerebellum posterior lobe	−21	−54	−60	5.1245	472
R cerebellum anterior lobe	42	−36	−30	4.8888	85
L medial frontal gyrus	0	54	30	−4.0665	70
sMCI vs. HC					
L cerebellum posterior lobe	−39	−54	−57	4.9316	82
L inferior temporal gyrus	−60	−42	−27	5.6828	398
R cerebellum anterior lobe	45	−33	−30	4.3503	78
L medial frontal gyrus	−3	48	30	−4.6899	113

The *x*, *y*, *z* coordinates is the primary peak locations in the MNI space. Cluster size > 100 voxels in ANOVA analysis, TFCE-FWE corrected, *p* < 0.05; Cluster size > 20 voxels in post-hoc test, TFCE-FWE corrected, *p* < 0.05; sMCI, stable mild cognitive impairment; pMCI progressive mild cognitive impairment; HC, healthy control; L, left; R, right.

### Effective connectivity analysis

**Figure 3:**
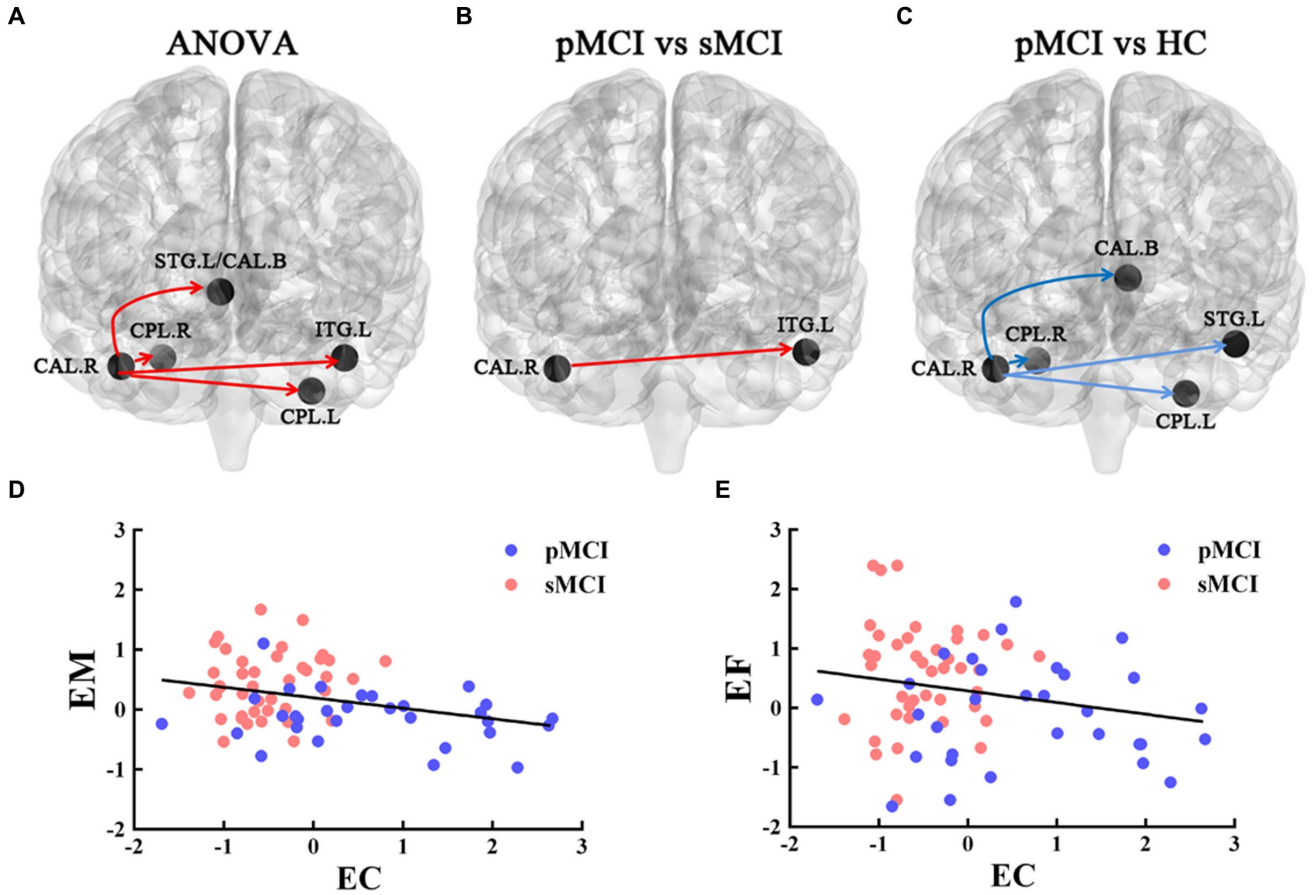
The EC of the right CAL. The red line represents the increased EC from right CAL to the other brain regions; the blue line represents decreased EC from right CAL to the other brain regions. **(A)** Brain region across three groups, including sMCI, pMCI, and HC (cluster size >100, *p* < 0.05). **(B,C)** Results of post-hoc analysis in voxel-wise analysis (TFCE-FWE corrected, cluster size >20, *p* < 0.05). **(D,E)** Significant associations between altered EC from right CAL to left ITG and cognitive function. sMCI, stable mild cognitive impairment; pMCI, progressive mild cognitive impairment; HC, healthy control; EC, effective connectivity; CAL, cerebellum anterior lobule; ITG, inferior temporal gyrus; EM, episodic memory; EF, executive function.

Furthermore, ANOVA revealed significant alterations in EC from the right CAL among the groups, encompassing the left bilateral CPL, bilateral superior temporal gyrus (STG), and bilateral caudate. Specifically, when compared with the HC group, sMCI demonstrated decreased EC from the right CAL to the bilateral CPL, bilateral caudate, and left STG. Additionally, when compared with sMCI, pMCI displayed increased EC from the right CAL to the left ITG (TFCE-FWE corrected, cluster size of >20, *p* < 0.05). These findings were consistent after controlling for age, sex, years of education, and gray matter volumes (Table 3 and Figure 3).

**Table 3:** The difference of effective connectivity from right CAL to brain regions across three groups.

Region (aal)	Peak MNI coordinate			*F*/*t*	Cluster number
	*x*	*y*	*z*		
ANOVA					
L cerebellum posterior lobe	−27	−54	−39	9.3355	506
L superior temporal gyrus/B Caudate	6	18	−3	9.0143	717
R cerebellum posterior lobe	27	−63	−27	9.4202	298
L inferior temporal gyrus	−39	−45	−27	8.6488	132
pMCI vs. sMCI					
L inferior temporal gyrus	−48	−27	−24	4.5613	27
sMCI vs. HC					
L cerebellum posterior lobe	−27	−54	−39	−4.3741	423
R cerebellum posterior lobe	27	−63	−27	−3.9863	86
L superior temporal gyrus	−45	9	−21	−3.5551	30
B Caudate	−6	12	3	−3.9396	41

The *x*, *y*, *z* coordinates is the primary peak locations in the MNI space. Cluster size > 100 voxels in ANOVA analysis, *p* < 0.05; Cluster size > 20 voxels in post-hoc test, TFCE-FWE corrected, *p* < 0.05; sMCI, stable mild cognitive impairment; pMCI progressive mild cognitive impairment; HC, healthy control; L, left; R, right; B, bilateral.

### Correlation analysis

The EC originating from the right CAL and extending to the left ITG was negatively associated with EM (*r* = −0.371, *p* = 0.002) and EF (*r* = −0.284, *p* = 0.018; Bonferroni corrected, *p* < 0.05; Figure 3).

### ROC analysis

**Figure 4:**
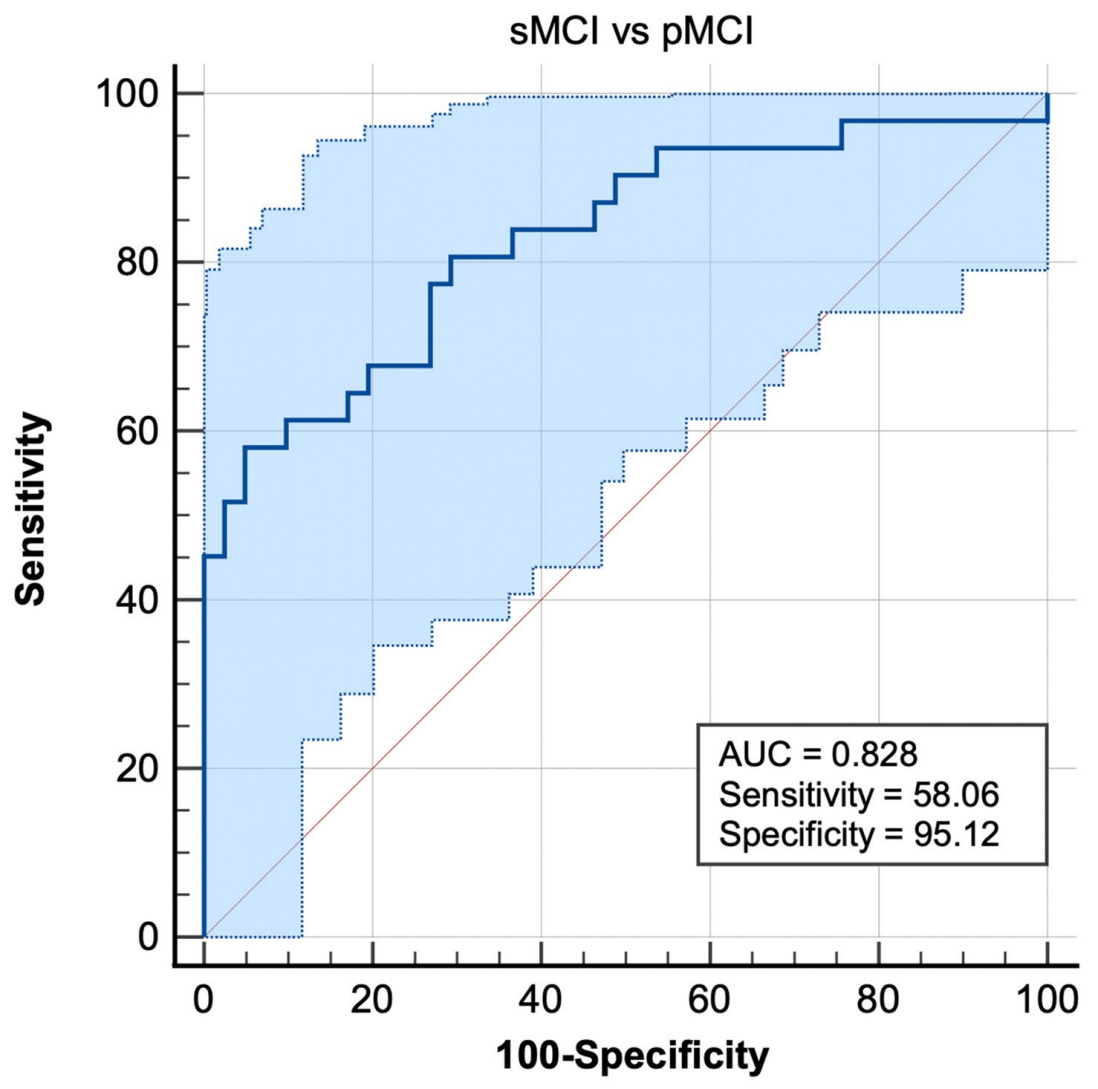
Combination of ROC analysis and EC from right CAL to left ITG differentiated pMCI from sMCI. sMCI, stable mild cognitive impairment; pMCI, progressive mild cognitive impairment; EC, effective connectivity; CAL, cerebellum anterior lobule; ITG, inferior temporal gyrus.

Most notably, EC from the right CAL to right ITG exhibited the ability to effectively distinguish sMCI from pMCI, with sensitivity, specificity, and accuracy of 0.5806, 0.9512, and 0.828, respectively (Figure 4).

## Discussion

To the best of our knowledge, the present study is the first to explore intrinsic brain functional hubs and causal connectivity in individuals with sMCI and pMCI, employing a combination of the DC and GCA methodologies. Using the DC analysis, we identified elevated DC in the left CPL, right CAL, and left ITG, along with reduced DC in the left MFG, in both sMCI and pMCI compared with HCs. Subsequently, the GCA was conducted, leveraging the altered DC values in an ANOVA setup to investigate their causal effect across the whole brain. Specifically, compared with HC participants, pMCI exhibited increased EC originating from the right CAL and extending to the left ITG, a connectivity pattern with a significantly negative association with impairments in EM and EF. Of particular significance, the EC extending from the right CAL to the right ITG demonstrated excellent discriminatory ability between sMCI and pMCI, together with high specificity and accuracy. This study delves into a more profound understanding of the underlying pathological mechanisms characterizing sMCI and pMCI.

The present study showed similar changes in DC values for sMCI and pMCI, shedding light on shared neuroimaging mechanisms between these two conditions. Both groups exhibited increased DC in the left ITG, left CPL, and right CAL, coupled with decreased DC in the left MFG. The coexistence of increased and diminished DC underlines a compensatory mechanism operating in the context of cognitive decline, a phenomenon previously substantiated by existing research (Xue C. et al., 2019; Xue et al., 2021). The left ITG plays a pivotal role in higher-order cognitive functions encompassing visual recognition, language comprehension, decision-making, and emotion regulation (Jackson et al., 2018). Notably, prior research identified the ITG as a hub for intense local β-amyloid/tau interactions, fostering a connectivity profile conducive to accelerated tau propagation (Lee et al., 2022). Moreover, diminished ReHo values in the left ITG in the MCI group were found to be correlated negatively with disease duration (Wu et al., 2022). These findings parallel those of Wang et al., who demonstrated significant alterations in nodal properties (degree and betweenness centrality) within the ITG in AD and MCI, thereby contributing to global topological alterations, consistent with our current study (Wang et al., 2018).

The human cerebellar cortex represents a complex structure characterized by intricate folding, surpassing even the cerebral cortex in this regard (Sereno et al., 2020). Its extensive neural fiber connections with the brain’s cognitive network underscore its pivotal role in shaping behavior and cognition evolution (Sereno et al., 2020). While the cerebellum primarily governs sensorimotor and vestibular functions, it additionally exerts influence over cognitive, emotional, and autonomic domains (Cutando et al., 2022). Notably, age-related declines in cerebellar lobular volumes and cortico-cerebellar FC have been linked to cognitive regression in healthy elderly individuals (Uwisengeyimana et al., 2020; Gellersen et al., 2021). Tang et al. proposed that cortico-cerebellar FC disruptions, particularly within the DMN and fronto-parietal networks, could serve as a novel avenue for early diagnosis and potential early interventions in MCI and AD (Tang et al., 2021). Furthermore, augmented cerebellar activity has been positively correlated with memory enhancement, potentially operating as a compensatory mechanism (McLaren et al., 2012). Hence, heightened connectivity in this context may potentially underpin cerebellar compensatory processes.

The MFG encompasses both caudal and rostral areas, with the latter encompassing portions of the dorsal lateral prefrontal cortex responsible for executive cognitive functions, thinking and perception, memory retrieval, problem-solving, and emotional regulation (Friedman and Robbins, 2022). A meta-analysis confirmed a significant association between reduced gray matter density, cerebral blood flow, hypometabolism in the MFG and increased anosognosia scores in patients with AD (Hallam et al., 2020). Caffarra et al. identified the left anterior cingulate and MFG as mediators of delayed free recall (Caffarra et al., 2016). Furthermore, diminished eigenvector centrality in the MFG was identified in MCI by Lou et al., aligning with the present study’s findings of reduced FC in this region (Lou et al., 2016). Importantly, it becomes evident that the alterations are primarily localized to the DMN, a network pivotal for self-referential psychological processes and social functions (Menon, 2023). It is the most studied network and is thought to be the first network with the risk of damage during AD because it is more susceptible to β-amyloid and tau deposition and glucose hypometabolism, which positions it as an early target in AD pathogenesis (Chiesa et al., 2019; Ingala et al., 2021). Our study corroborates the DMN’s vulnerability as a leading disrupted network in MCI.

Intriguingly, significant differences in DC were not discernible between sMCI and pMCI. However, pronounced distinctions emerged in EC, indicating that EC might offer nuanced neuroimaging insights. Compared with HCs, sMCI displayed a marked EC reduction extending from the right CAL to the bilateral CPL, left STG, and bilateral caudate. The caudate nucleus plays a key role in various higher neural functions, instrumental in executive functions, learning, memory, motivation, and emotion, and it assumes a multifaceted role in higher-order neural functions (Grahn et al., 2008; Yang et al., 2022). The cortical-caudate FC was less distinct in older adults versus younger counterparts, with age-related differences in caudate function associated with memory decline in the context of normal aging (Rieckmann et al., 2018). Task-based fMRI data unveiled notable caudate activity in older adults during virtual navigation tasks, in contrast to hippocampal engagement observed in younger adults, implying an age-related shift in functional demands from the hippocampus to the caudate nucleus during navigation (Konishi et al., 2013). The caudate volume was lower in patients with MCI than in HC participants (Madsen et al., 2010). Gao et al. reported reduced regional homogeneity in the right caudate of patients with MCI relative to HC participants, suggesting that right caudate ReHo can be used as a neuroimaging biomarker of MCI, which can provide objective guidance for the diagnosis and management of MCI in the future (Gao et al., 2022).

Interestingly, no significant difference in EC existed between pMCI and HC, indicating that altered EC could potentially characterize sMCI. Zhou et al. found that sMCI cortical thickness decreased (e.g., ITG and anterior cingulated regions) and node and network efficiency increased, revealing the coexistence of injury and compensation (Zhou and Lui, 2013). The decrease in EC from the right CAL to other regions in sMCI may be a specific network change in sMCI. Notably, pMCI showcased elevated EC extending from the CAL to ITG compared with sMCI. Previous studies underscored compromised FC between the cerebellum and the cerebral cortex (Olivito et al., 2020; Zhou Z. et al., 2021). The results further corroborate the notion that AD/MCI entails disrupted mechanisms influencing synaptic plasticity in long-range interconnections across brain regions (Lou et al., 2016; Sha et al., 2018). Correlation analysis showed that there was a negative correlation between EC from CAL to ITG and EM scores, wherein EC from CAL to ITG increased with the decline of EM, reinforcing the compensatory hypothesis (Gaubert et al., 2019; Bhembre et al., 2023). This phenomenon likely reflects the brain’s endeavor to counteract cognitive decline by augmenting neural activity or connectivity. Of paramount importance, the EC extending from CAL to ITG can effectively discriminate sMCI from pMCI with high specificity and accuracy. Collectively, increased EC originating in the cerebellum and extending to the temporal lobe serves as a promising biomarker for the differentiation and diagnosis of sMCI and pMCI, offering a new perspective for therapeutic target selection.

## Limitations

There were three limitations to our study. First, there was a significant difference in years of education across the three groups, which could have potentially confounding our results. However, to mitigate these confounding effects, all statistical analyses were conducted with age, years of education, and gender as covariates. As such, we maintain the credibility of our findings. Secondly, the small sample size of our study may affect the robustness and repeatability of the study. We used strict multiple comparisons to ensure reliability. We will further validate the results when the sample size is expanded in the future. Thirdly, important biomarkers such as APOE-e4 carrier status, Aβ deposition, and tau tangles deposition were not statistically analyzed, which were crucial in the research of the pathological mechanism of MCI. In the following studies, we will explore the value of imaging indicators in predicting MCI disease transformation and pathological features.

## Conclusion

In summary, this study uncovers shared and distinctive alterations in DC and EC between sMCI and pMCI, with implications for cognitive function. Importantly, the unidirectional EC disruptions stemming from the cerebellum and extending to the temporal lobe hold considerable potential for effectively distinguishing sMCI from pMCI while also offering novel insights into the neural circuit mechanisms linking the temporal lobe and cerebellum in MCI.

## Data Availability

The datasets presented in this study can be found in online repositories. The names of the repository/repositories and accession number(s) can be found in the article/Supplementary material.

## Footnotes

^1^ http://adni.loni.usc.edu

^2^ http://adni.loni.usc.edu/data-samples/access-data/

^3^DPABI, http://rfmri.org/DPABI.

^4^ http://www.mathworks.com/products/matlab/

^5^rs-HRF, https://www.nitrc.org/projects/rshrf.

## Group member of the Alzheimer’s Disease Neuroimaging Initiative

Data used in preparation of this article were obtained from the Alzheimer’s Disease Neuroimaging Initiative (ADNI) database (adni.loni.usc.edu). As such, the investigators within the ADNI contributed to the design and implementation of ADNI and/or provided data but did not participate in analysis or writing of this report. A complete listing of ADNI investigators can be found at: http://adni.loni.usc.edu/wp-content/uploads/how_to_apply/ADNI_Acknowledgement_List.pdf.
