# Supplementary material for: Alteration in temporal-cerebellar effective connectivity can effectively distinguish stable and progressive mild cognitive impairment
Source: Front Aging Neurosci. 2024 Aug 29;16:1442721. doi: 10.3389/fnagi.2024.1442721 (PMC11390694; doi:10.3389/fnagi.2024.1442721)
Supplement: Supplementary file 1 [file Table_1.DOCX]

**Alteration in temporal-cerebellar effective connectivity can effectively distinguish stable and progressive mild cognitive impairment**

Chen Xue, Darui Zheng, Yiming Ruan, Wenxuan Guo, Jun Hu^*^, and the Alzheimer’s Disease Neuroimaging Initiative^‡^

Department of Radiology, the Affiliated Brain Hospital of Nanjing Medical University, Nanjing, 210029, China

^‡^Data used in preparation of this article were obtained from the Alzheimer’s Disease Neuroimaging Initiative (ADNI) database (adni.loni.usc.edu). As such, the investigators within the ADNI contributed to the design and implementation of ADNI and/or provided data but did not participate in analysis or writing of this report. A complete listing of ADNI investigators can be found at: <http://adni.loni.usc.edu/wp-content/uploads/how_to_apply/ADNI_Acknowledgement_List.pdf>

***Running Title:*** altered EC in MCI.

***Correspondence to:**

**Jun Hu**, Department of Radiology, the Affiliated Brain Hospital of Nanjing Medical University, No.264, Guangzhou Road, Gulou District, Nanjing, Jiangsu, 210029, China. Email: njnkyyhujun@163.com

**Materials and methods**

**Participants**

The inclusion of MCI patients were based on the ADNI-2 procedures manual: 1) memory complaint; 2) Abnormal memory function documented by scoring within the education adjusted ranges on the Logical Memory II subscale (Delayed Paragraph Recall, Paragraph A only) from the Wechsler Memory Scale Revised (≤ 8 for 16 or more years of education; ≤ 4 for 8-15 years of education; ≤ 2 for 0-7 years of education); 3) Clinical Dementia Rating (CDR) = 0.5; 4) Mini-Mental State Examination (MMSE) scores between 24 and 30; 5) no dementia and no signal of depression (Geriatric Depression Scale, GDS < 6). The inclusion of AD patients were based on the ADNI-2 procedures manual: 1) memory complaint; 2) Abnormal memory function documented by scoring below education adjusted cutoffs on the Logical Memory II subscale (Delayed Paragraph Recall, Paragraph A only) from the Wechsler Memory Scale–Revised (≤ 8 for 16 or more years of education; ≤ 4 for 8-15 years of education; ≤ 2 for 0-7 years of education), MMSE between 20 and 24; 3) CDR = 0.5 or 1.0. The inclusion of HC were: 1) no memory complaints; 2) normal cognitive performance, MMSE between 24 and 30, and GDS < 6; 3) CDR = 0.

**Neuropsychological Assessment**

EM was evaluated by the composite score of the Rey Auditory Verbal Learning Test, the Alzheimer Disease Assessment Scale-Cognitive, Logical Memory, and MMSE. EF was assessed by the composite score of Category Fluency, WAIS-R Digit Symbol, Trails A & B, Digit Span Backwards, and clock drawing.

**Functional data preprocessing**

The preprocessing steps were as follows: (1) discarding the first 10 functional image volumes; (2) correcting slice timing and head motion; participants with excessive head motion (cumulative translation or rotation of >3.0 mm or 3.0°) were excluded; (3) spatially normalizing images to the Montreal Neurological Institute (MNI) echo-planar imaging template and resampled to 3 × 3 × 3 mm^3^ voxels; (4) removing nuisance variables such as 24 head motion parameters, global mean signal, white matter signal, and cerebrospinal fluid signal; (5) smoothing normalized brain volumes with a Gaussian kernel of 6-mm full-width half-maximum to reduce individual variations; (6) band-pass filtering with frequencies between 0.01 and 0.08 Hz.

**DC analysis**

Pearson's correlation coefficients (r) were computed between the time course of a given voxel and all other whole-brain voxels within the whole-brain gray matter mask. An undirected adjacency matrix was generated by thresholding correlations at r > 0.25 [25]. DC was determined as the sum of the weights of the effective connections (weighted) for each voxel, divided by the mean whole-brain DC value to yield a standardized DC value. Lastly, to improve data normality, the correlation coefficient was converted to Z values through Fisher Z transformation, which was used for further analysis.
